# Community responses of arbuscular mycorrhiza fungi to hydrological gradients in a riparian *Phragmites australis* wetland

**DOI:** 10.1002/ece3.11271

**Published:** 2024-04-11

**Authors:** Xue‐dong Chen, Ying Zhu, Mei‐na Feng, Ji‐hang Li, Ming‐yan Shi

**Affiliations:** ^1^ College of Life Science Luoyang Normal University Luoyang China; ^2^ West Henan Yellow River Wetland Ecosystem Observation and Research Station Luoyang Normal University Luoyang China

**Keywords:** arbuscular mycorrhiza fungi, community, moisture, wetland

## Abstract

The hydrological regime is considered to be the major factor that affects the distribution of arbuscular mycorrhiza (AM) fungi in wetlands. We aimed to investigate the responses of AM fungal community to different hydrological gradients. Illumina Miseq sequencing technology was used to study the AM fungal community structure in roots and rhizosphere soils of *Phragmites australis* in different moisture areas (dry area, alternating wet and dry area, and flooded area) in Mengjin Yellow River wetland. The rhizosphere soils and roots hosted different AM fungal communities. In roots, the AM fungal colonization and Chao1 richness in dry area were significantly higher than that in alternating wet and dry area and flooded area, but the community composition did not vary clearly under different water conditions. In rhizosphere soils, the Chao1 richness of AM fungi in flooded area was significantly higher than that in alternating wet and dry area and dry area, and the AM fungal community structure obviously differed across different areas. The redundancy analyses indicated that changes in the AM fungal community in soils were associated with altered soil properties, and the abundance of the dominant genus *Glomus* was mostly positively correlated with alkali‐hydrolyzable nitrogen in soils. This study helps us to understand the responses of AM fungal community to hydrological gradients in wetlands.

## Graphical abstract

AM fungal community in rhizosphere soils of *Phragmites australis* exhibited obvious variation under different hydrological gradients. AM fungal community in *Phragmites australis* roots were not significantly affected by water regime.

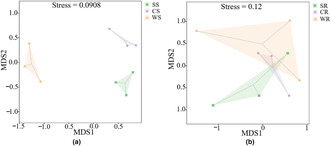

## 1 INTRODUCTION

Arbuscular mycorrhiza (AM) fungi are probably the most widespread soil microorganisms, which can form symbiosis with most plants, and are the key node connecting the aboveground and underground ecosystems. Numerous studies have shown that AM fungi play an important role in the terrestrial ecosystem, with various functions such as improving plant growth and nutrient absorption (Bhantana et al., 2021), enhancing plant resistance to biotic and abiotic stresses (Amir et al., 2013), promoting nutrient geochemical cycle (Craig et al., 2018), improving soil structure (Lehmann et al., 2017) and repairing soil pollution (Solís‐Ramos et al., 2021).

Wetlands are well known as the kidney of the earth with abundant biological resources and ecological functions such as purifying the water source, mitigating floods and droughts, and regulating regional microclimate (Chakraborty et al., 2023; Ye et al., 2022). In recent years, increasing numbers of reports have confirmed that AM fungi also exist widely in wetland habitats and can colonize the roots of various hygrophytes to form AM symbiotic structure (Xu et al., 2016; Yan et al., 2008). Previous studies on AM fungi in wetlands focused mainly on promoting the growth and nutrient absorption of wetland plants (Hu et al., 2020; Liang et al., 2018; Miller & Sharitz, 2000), but little attention is paid to AM fungal community composition, ecological functions, and related environmental influencing factors.

Nowadays, due to social, economic, and global climate change as well as human overexploitation, wetlands have been seriously damaged. Deterioration of the ecological environment has caused significant differences in water content in different habitats of wetland. In terrestrial ecosystem, the formation and growth of AM fungi are mainly affected by vegetation types (Kiers et al., 2011), soil properties (Higo et al., 2018), climate, and other factors (de Souza & Santos, 2018; Kivlin et al., 2011). Compared to the terrestrial ecosystem, soil moisture and soil oxygen availability directly affected by the water regime are the main factors limiting the development of AM fungi in wetlands (Daleo et al., 2007; Stevens et al., 2011; Wang et al., 2016). Some studies found an increase in the intensity of AM fungal colonization with increased water levels (Brown & Bledsoe, 1996; Wang et al., 2015), while others reported a decrease (Hu et al., 2020; Miller, 2000; Wang et al., 2011) or no relationship (Ipsilantis & Sylvia, 2007; Miller & Sharitz, 2000). Bickford et al. (2018) observed no structures characteristic of AM fungi in *P. australis* roots under unsaturated, saturated, or submerged conditions, and thought that high water levels creating anaerobic soil conditions would make it difficult for germinating spores of AM fungi to reach the root surface. These results have not been conclusive, and these studies focused only on the colonization status of AM fungi but did not include research at the community level. So the influence of water level on the community composition and diversity of AM fungi in wetlands and its mechanism are still unclear. The species types and population of AM fungi are closely related to their functions, directly affecting the stability and health of the environmental ecosystem (Jiang et al., 2018). The rich AM fungal community is more adaptable to environmental changes and achieves synergistic effects of different functions. Therefore, studying AM fungal presence, distribution and response patterns under different water conditions could increase our understanding of AM fungal community structure in wetlands, and also provide insights into the effects of hydrological gradients on shaping such communities.

In this study, the roots and rhizosphere soils of *P. australis* were sampled in habitats with different water conditions (dry area, alternating wet and dry area, flooded area) in Mengjin Yellow River Wetland in China. Subsequently, the physical and chemical properties of the rhizosphere soil were determined, and the composition of AM fungal community in rhizosphere soils and roots were also identified. The differences in the distribution of AM fungal species in habitats and their relationships with soil properties were then analyzed. The purposes of this study were: (i) to explore the responses of AM fungal community to different hydrological alterations, and (ii) to reveal other relevant soil influencing factors driving the assembly of AM fungal community.

## 2 MATERIALS AND METHODS

### 2.1 Experimental site and sample collection

This study was carried out in November 2020 at the experimental site of Mengjin Yellow River Wetland Field Observation Station (34°47′ N, 112°29′  E), in the north of Kouma Village, Henan Province, China. As the boundary between the middle and lower reaches of the Yellow River, it has a special ecological status. This region belongs to the warm temperate monsoon climate, with an annual average temperature of 13.7°C and an annual average rainfall of 650.2 mm. It has a large area of natural *P. australis* vegetation. Influenced by human activity disturbances in recent years, the water content in different habitats of the wetland is quite different, forming a natural perennial dry area, a perennial flooded area, and an alternating wet and dry area.

**FIGURE 1:**
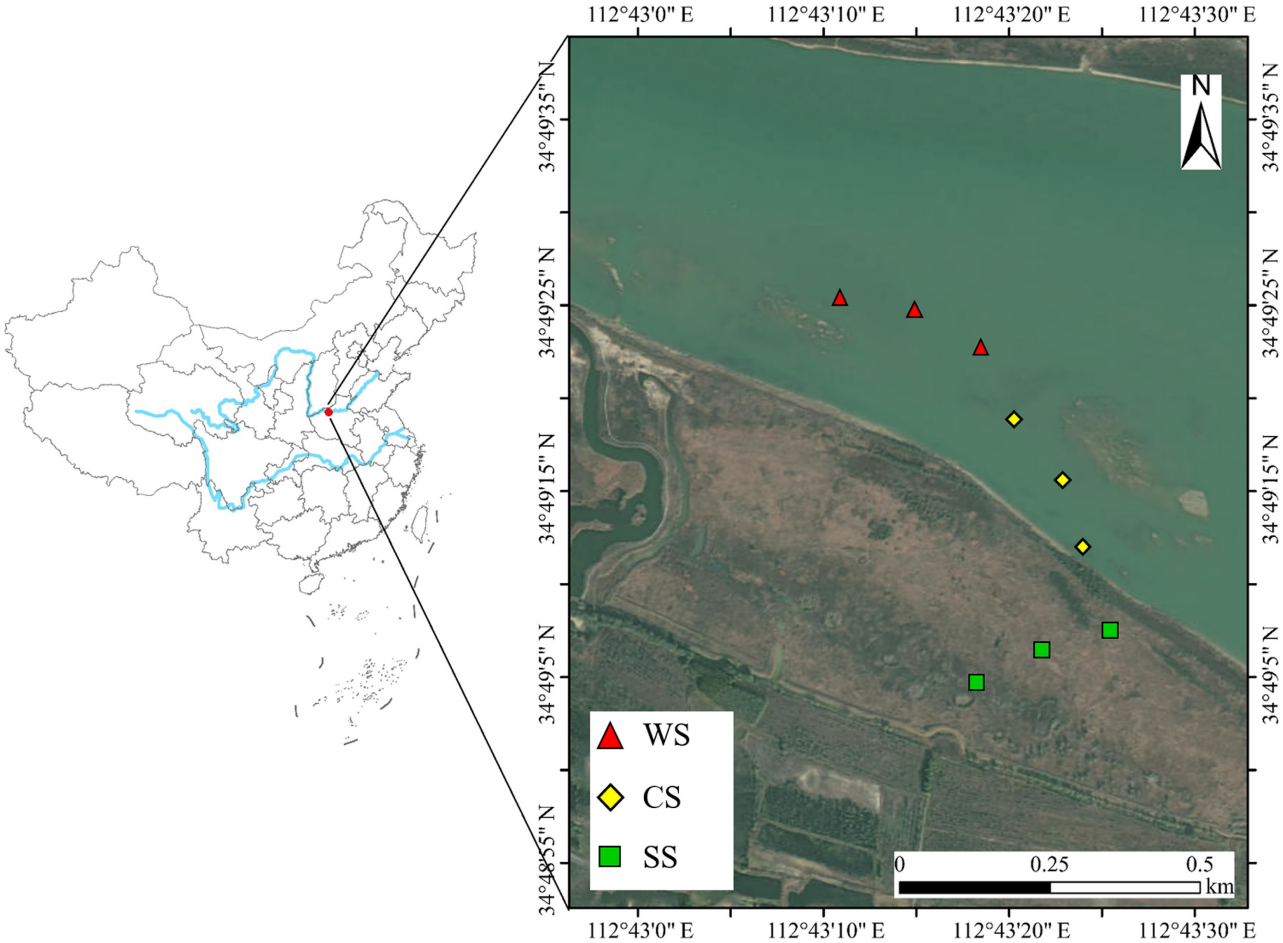
Distribution of sampling points. CS, alternating wet and dry area; SS, dry area; WS, flooded area.

The *P. australis* habitats with different water levels (dry area, alternating wet and dry area, flooded area) were selected as sample areas (Figure 1), and the selected sampling areas had similar background conditions such as soil, climatic and geographic characteristics. We randomly established three duplicate sample plots of 20 m × 20 m in each area, and the three sample plots within the same area were well separated by a distance of more than 100 m between two adjacent plots. Then five *P. australis* were selected in each sampling plot, and the rhizosphere soils and roots of *P. australis* were sampled. Next, the five samples from *P. australis* were mixed as a representative sample of one plot and transported back to the laboratory for further analysis.

### 2.2 Soil properties

pH was measured using a 1:2.5 soil/solution ratio in distilled water (Hendershot et al., 1993). Total organic carbon was measured with 1 g sample of dry‐sieved 0.15 mm soil using the potassium dichromate volumetric method (Bao, 2000). The available K was determined with 5 g sample of dry‐sieved 1 mm soil using CH_3_COONH_4_ extraction flame spectrophotometry (Bao, 2000). The available P was quantified with 1 g sample of dry‐sieved 1 mm soil using the molybdenum antimony colorimetric method (Bao, 2000). The alkali‐hydrolyzable N was measured with 1 g sample of dry‐sieved 1 mm soil by using the alkaline hydrolysis nitrogen diffusion method (Bao, 2000).

### 2.3 Glomalin content

Total glomalin (TG) and easily extractable glomalin (EEG) were determined according to Wright and Upadhyaya (1996). Replicate 0.25 g samples of dry‐sieved 1 mm soil were extracted with 2 mL of extractant. TG was extracted with 50 mM citrate (pH 8.0) at 121°C. EEG was extracted with 20 mM citrate, pH 7.0 at 121°C for 30 min. The time required was 90 min for all samples. For sequential extractions, the supernatant was removed by centrifugation at 10,000 *g* for 5 min. 2 mL of 50 mm citrate (pH 8.0) was added to the residue, and samples were autoclaved for 60 min. Extraction of a sample continued until the supernatant showed none of the red‐brown color typical of glomalin. Extracts from each replicate were pooled and then analyzed. After extraction cycles were completed, samples were centrifuged to remove the soil particles (10,000 *g* for 5 min), and protein in the supernatant was determined by the Bradford dye‐binding assay with bovine serum albumin as the standard.

### 2.4 AM fungal colonization

5 g fresh roots was cleared in 10% KOH at 90°C for 30 min, bleached in alkaline H_2_O_2_ for 20 min, acidified in 1% HCl for 3 min, stained in lactophenol trypan blue, and finally discolored with a 50%(v/v) glycerol solution (Phillips & Hayman, 1970). The colonization level was estimated using the gridline intersect method described by Giovannetti and Mosse (1980).

### 2.5 DNA extraction, PCR, and sequencing

Genomic DNA was extracted, respectively, from 1 g fresh rhizosphere soil samples and 0.5 g root samples using the Soil DNA Kit and the Plant DNA Kit (Omega Biotek) and stored at −20°C prior to further analysis. The Glomeromycotan ribosomal small subunit (SSU) was amplified using the forward primer AMV4.5NF (5′‐AAGCTCGTAGTTGAATTTCG‐3′) and the reverse primer AMDGR (5′‐CCCAACTATCCCTATTAATCAT‐3′) (Lumini et al., 2010). The 25‐μL PCR reaction mixtures contained 5× reaction buffer (5 μL), 5×GC buffer (5 μL), dNTP (2.5 mM, 2 μL), forward primer (10 μM, 1 μL), reverse primer (10 μM, 1 μL), DNA template (2 μL), ddH_2_O (8.75 μL), and Q5 DNA polymerase (0.25 μL). The amplification parameters were as follows: 98°C for 2 min, followed by 25 cycles at 98°C for 15 s; at 55°C for 30 s; at 72°C for 30 s, and a final extension at 72°C for 5 min. The PCR products were purified and then sequenced on the Illumina Miseq platform in Shanghai Personal Biotechnology Co., Ltd. All raw sequences were deposited in the NCBI Sequence Read Archive under accession number SRP440790.

### 2.6 Sequence analysis

Microbiome bioinformatics was performed with QIIME2 (Bolyen et al., 2018). Raw sequence data were demultiplexed and then quality filtered, denoised, and merged using the DADA2 plugin (Callahan et al., 2016). Sequences processed were clustered into operational taxonomic units (OTUs) defined by 97% similarity, and 18S reads were cleaned according to the MaarjAM database (Öpik et al., 2010). Non‐singleton OTUs were aligned with MAFFT and rarefied to ensure further analysis at the same level for every sample (Zhan et al., 2023). OTU‐level alpha diversity metrics (Chao1, Shannon, Simpson, Pielou's evenness), and beta diversity analysis metrics (Bray–Curtis dissimilarity) were estimated using the diversity plugin (Chen et al., 2021; Shannon, 1997).

### 2.7 Statistical analysis

The differences in soil physical–chemical properties, glomalin contents, AM fungal colonization under different hydrological conditions were analyzed by one‐way analysis of variance using SPSS 22.0 software. Data were visualized using Origin 9.0 software. Venn diagram was generated by using R package “VennDiagram” to visualize the shared and unique OTUs among samples. Box plots were drawn to compare the OTU‐level alpha diversity metrics among samples. For normally distributed alpha diversity metrics, ANOVA with Duncan's significance test was used to perform pairwise comparisons. The differences in the composition of AM fungal community were compared by principal coordinates analysis (PCoA) complemented with Permanova permutational multivariate analysis of variance based on the Bray–Curtis dissimilarity distances and visualized using R package “ggplot2.” Redundancy analysis (RDA) was performed to explore the relationships between the soil properties and AM fungal community in Canoco 4.5.

## 3 RESULTS

### 3.1 Soil properties

There was a significant difference in the content of alkali‐hydrolyzable N (*p* < .05). The alkali‐hydrolyzable N content in flooded area was 8.84 mg/kg, which was the highest, while the lowest with 5.77 mg/kg in dry area. Additionally, the contents of total organic carbon (3.86 g/kg), available K (89.50 mg/kg), and available P (4.11 mg/kg) in flooded area were also higher than those in dry area or alternating wet and dry area, although there was no statistically significant difference (Table 1).

**TABLE 1:** Rhizosphere soil properties of *Phragmites australis* under different water conditions.

	pH	Total organic carbon (g/kg)	Available K (mg/kg)	Alkali‐hydrolyzable N (mg/kg)	Available P (mg/kg)
SS	8.42 ± 0.09a	1.51 ± 0.09a	47.00 ± 7.76a	5.77 ± 0.41b	2.09 ± 0.29a
CS	8.40 ± 0.15a	2.16 ± 0.29a	51.33 ± 6.33a	6.83 ± 0.54ab	2.75 ± 0.09a
WS	8.41 ± 0.09a	3.86 ± 2.06a	89.50 ± 47.15a	8.84 ± 1.87a	4.11 ± 1.70a
*p* Value	.923	.120	.201	.044	.113

*Note*: Different lowercase letters represent significant differences in Duncan test at 0.05 level (mean ± SE, *n* = 3).

Abbreviations: CS, rhizosphere soil of *P. australis* in alternating wet and dry area; SS, rhizosphere soil of *P. australis* in dry area; WS, rhizosphere soil of *P. australis* in flooded area.

### 3.2 Glomalin content in soils

**FIGURE 2:**
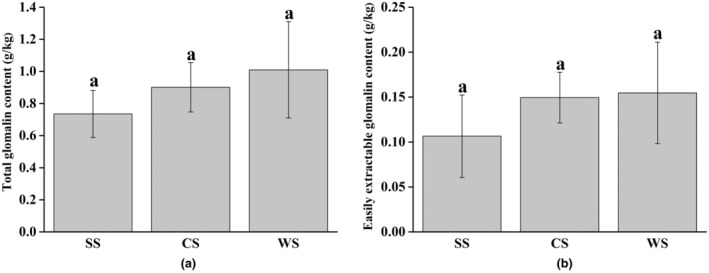
The contents of total glomalin (a) and easily extractable glomalin (b) extracted from rhizosphere soil of *P. australis* under different water conditions. CS, rhizosphere soil of *P. australis* in alternating wet and dry area; SS, rhizosphere soil of *P. australis* in dry area; WS, rhizosphere soil of *P. australis* in flooded area. Different lowercase letters represent significant differences in Duncan test at 0.05 level; mean ± SE, *n* = 3.

The contents of total glomalin and easily extractable glomalin in flooded area were, respectively, 1.01 and 0.15 g/kg, which were higher than those in dry area and alternating wet and dry area; however, the difference was not significant (Figure 2).

### 3.3 AM fungal colonization

**FIGURE 3:**
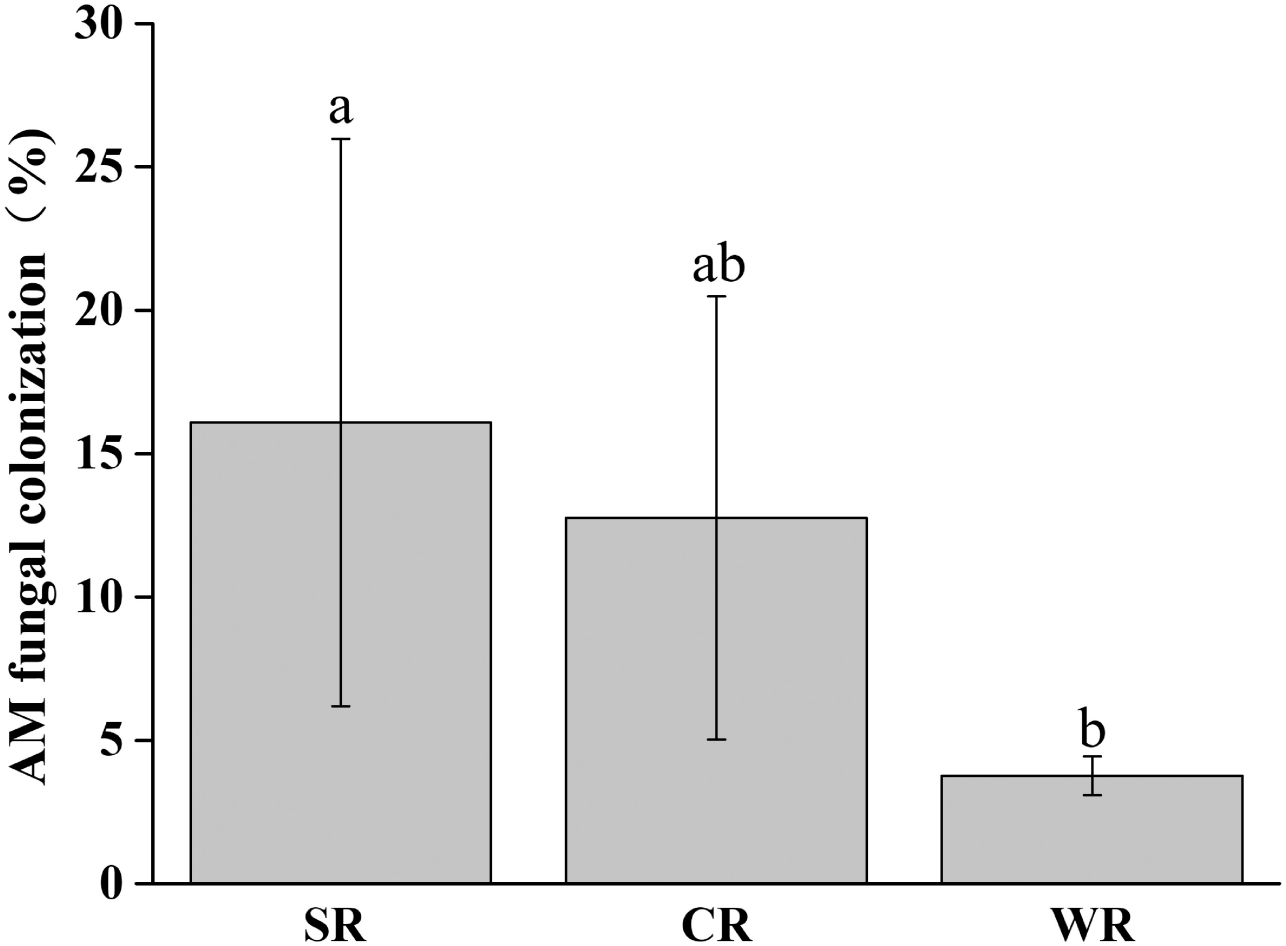
AM fungal colonization in *P. australis* roots under different water conditions. CR, roots of *P. australis* in alternating wet and dry area; SR, roots of *P. australis* in dry area; WR, roots of *P. australis* in flooded area. Different lowercase letters represent significant differences in Duncan test at 0.05 level; mean ± SE, *n* = 3.

The results showed that *P. australis* was colonized by AM fungi under any water conditions. The water level had a significant effect on AM fungal colonization. The AM fungal colonization rates in dry area, alternating wet and dry area, and flooded area were 16.08%, 12.76%, and 3.76%, respectively (Figure 3).

### 3.4 AM fungal community composition

**FIGURE 4:**
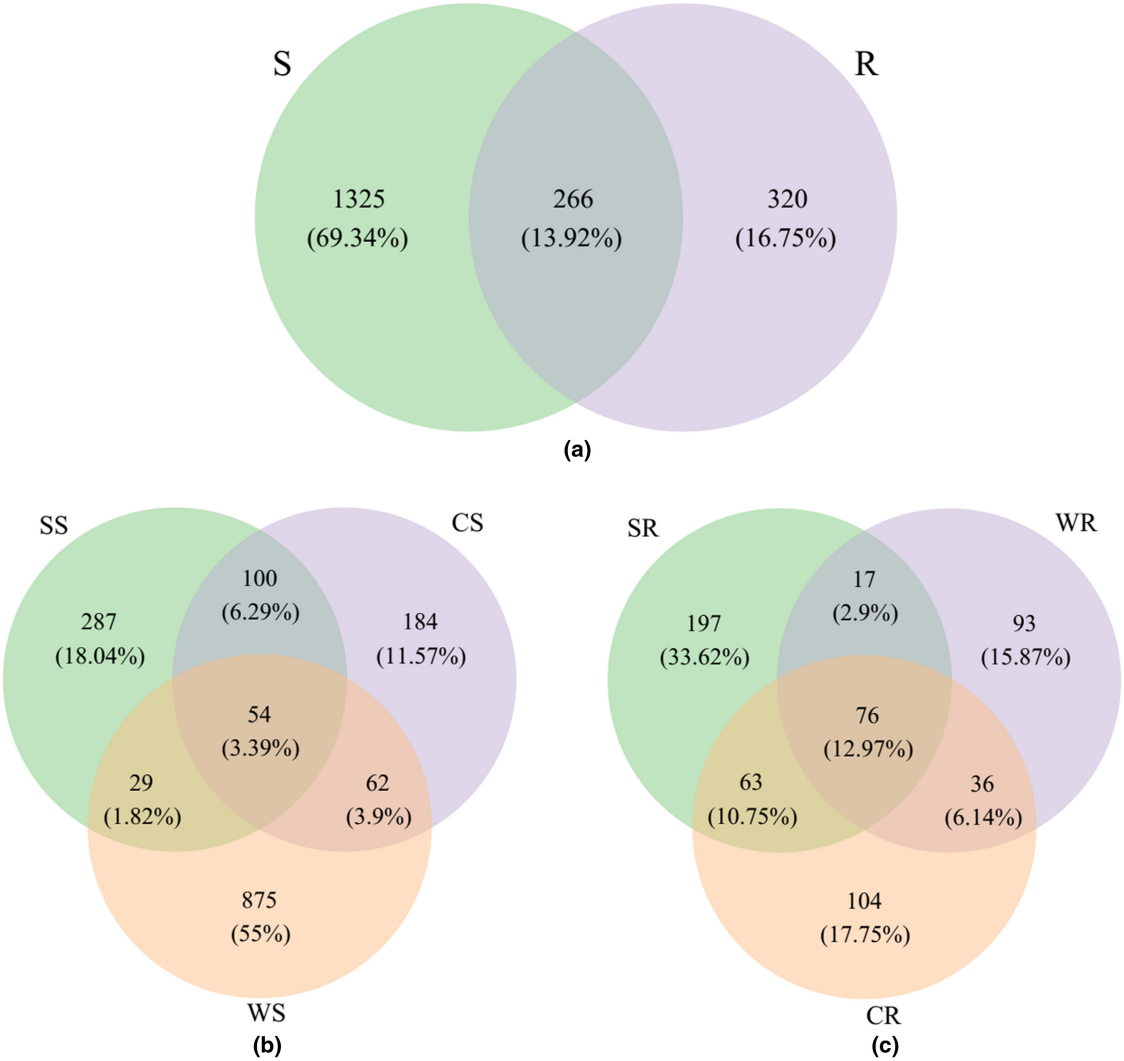
The venn diagrams showing the number and proportion of unique and shared OTUs between (a) rhizosphere soils and roots, (b) rhizosphere soils under different water conditions, and (c) roots under different water conditions. CR, roots of *P. australis* in alternating wet and dry area; CS, rhizosphere soil of *P. australis* in alternating wet and dry area; R, roots of *P. australis*; S, rhizosphere soil of *P. australis*; SR, roots of *P. australis* in dry area; SS, rhizosphere soil of *P. australis* in dry area; WR, roots of *P. australis* in flooded area; WS, rhizosphere soil of *P. australis* in flooded area. The same below.

After quality filtering, a total of 2,300,465 AM fungal sequences with an average length of 215 bases were obtained from nine soil samples and nine root samples. The rarefaction curve has indicated that the sequencing effort was enough to represent AM fungal diversity, since the curve has reached the platea (Figure S1). A total of 1916 AM fungal operational taxonomic units (OTU) were found. 1325 OTUs were unique to rhizosphere soils, dominated by *Glomus*, *Paraglomus*, *Claroideoglomus*, *Archaeospora*, *Scutellospora*, *Ambispora*. 320 OTUs were unique to roots, dominated by *Glomus*, *Paraglomus*, *Acaulospora*. 266 OTUs were shared between rhizosphere soils and roots, dominated by *Glomus*, *Paraglomus*, *Claroideoglomus*, *Diversispora*, *Ambispora* (Figure 4a, Figure S2). In rhizosphere soils, the number of unique OTU was the highest, reaching 875 in flooded area, and 184 unique OTUs were detected in alternating wet and dry area, and 287 unique OTUs in dry area (Figure 4b). In roots, the number of unique OTUs was the largest, up to 197 in dry area, and 104 unique OTUs were detected in alternating wet and dry area, and 93 unique OTUs in flooded area (Figure 4c).

**FIGURE 5:**
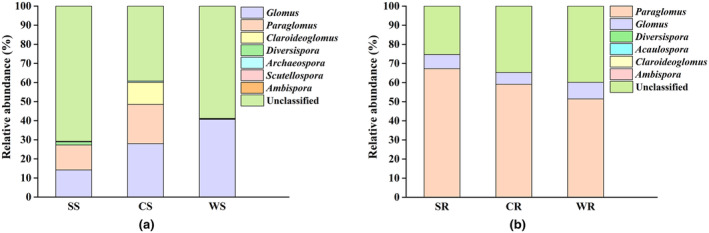
Changes in the composition of AM fungal community at the genus levels. (a) rhizosphere soil; (b) roots.

Through the identification of AM fungi in rhizosphere soils of *P. australis*, a total of 7 genera were found. There were 6 genera in dry area, of which the abundances of *Glomus* and *Paraglomus* were relatively high, accounting for 14.25% and 13.03%, respectively, followed by *Diversispora* (1.61%), *Scutellospora* (0.35%), *Claroideoglomus* (0.02%), and *Ambispora* (0.001%). There were also 6 genera in alternating wet and dry area, with *Glomus* accounting for 28.01%, *Paraglomus* for 20.55%, *Claroidoglomus* for 11.58%, *Diversispora* for 0.03%, *Archaeospora* for 0.58%, and *Ambispora* for 0.002%. Only 3 genera were found in flooded area, of which the proportion of *Glomus* was the highest, reaching 40.74%, followed by *Paraglomus* (0.22%) and *Claroideoglomus* (0.27%). In general, *Glomus* was the dominant genus in rhizosphere soils of *P. australis* (Figure 5a).

Through the identification of AM fungi in the roots of *P. australis*, a total of six genera were found. There were six genera in dry area, of which the abundance of *Paraglomus* was the highest, accounting for 67.33%, followed by *Glomus* (7.35%), *Diversispora* (0.07%), *Acaulospora* (0.02%), *Claroideoglomus* (0.004%), and *Ambispora* (0.002%). There were three genera in alternating wet and dry area, with *Paraglomus* accounting for 59.13%, followed by *Glomus* (6.21%) and *Claroideoglomus* (0.002%). Only two genera were found in flooded area, of which the abundance of *Paraglomus* was the highest (51.48%), followed by *Glomus* (8.69%). In general, *Paraglomus* was the dominant genus in roots of *P. australis* (Figure 5b). However, there were no significant differences in the relative abundances of *Paraglomus* and *Glomus* in roots and rhizosphere soils of *P. australis* in different water areas (Figure S3).

### 3.5 AM fungal diversity

**FIGURE 6:**
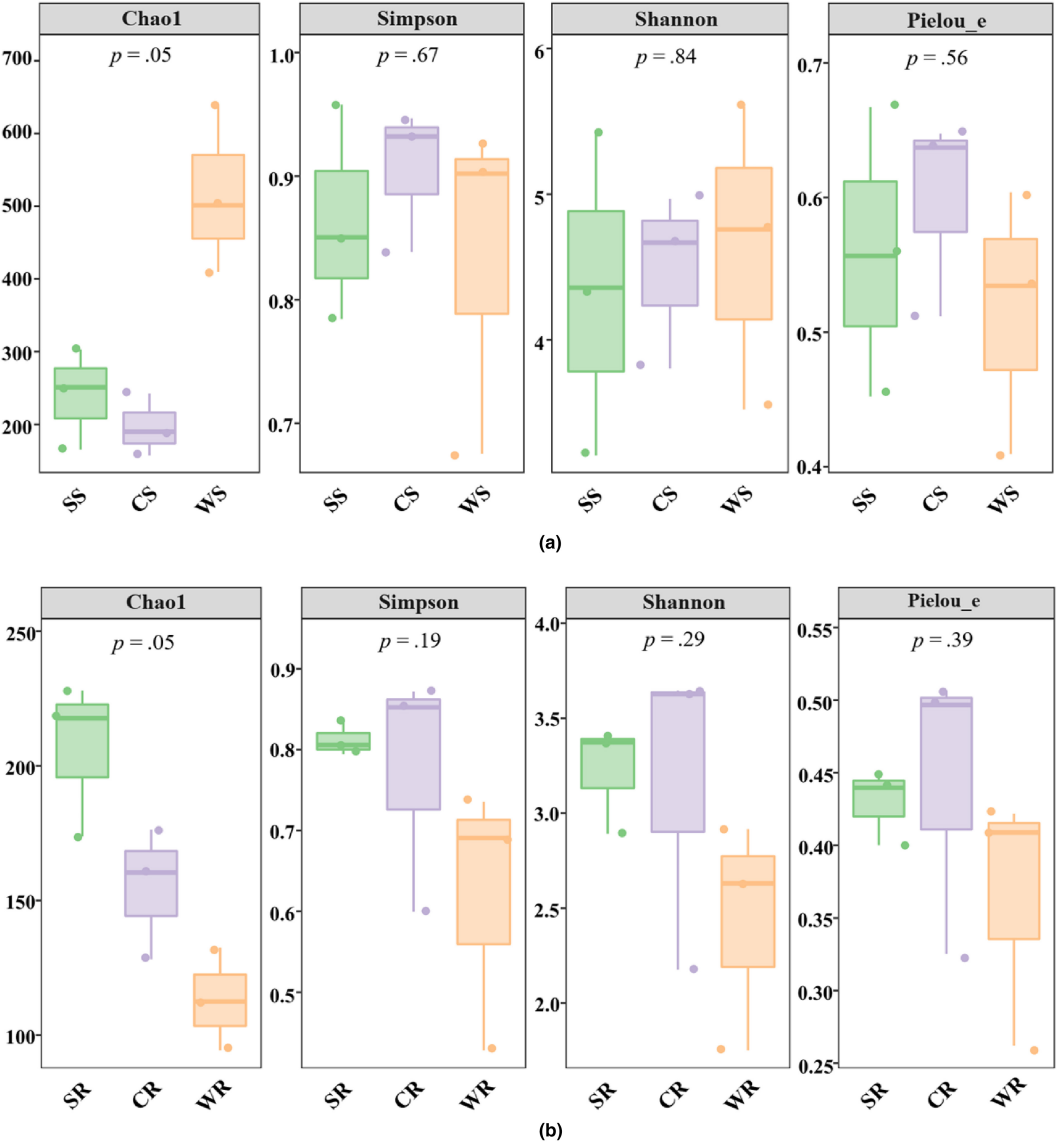
Boxplots of AM fungal diversity index in rhizosphere soils and roots of *P. australis* under different water conditions. (a) rhizosphere soils; (b) roots.

Based on the results of the AM fungal diversity index in rhizosphere soils, the Chao1 index of AM fungi in flooded area was 516.81, which was significantly higher than that in alternating wet and dry area, and dry area. The distributions of Simpson index, Shannon index, and Pielou_e index were not centralized, and the data were scattered in flooded area and dry area (Figure 6a). Inconsistently, in roots, the Chao1 index of AM fungi in dry area was 206.47, which was significantly higher than that in alternating wet and dry area, and flooded area. Moreover, the Simpson index, Shannon index, and Pielou_e index in flooded area were lower than that in dry area, and alternating wet and dry area. The distributions of Simpson index, Shannon index, and Pielou_e index in alternating wet and dry area, and flooded area were relatively scattered (Figure 6b).

**FIGURE 7:**
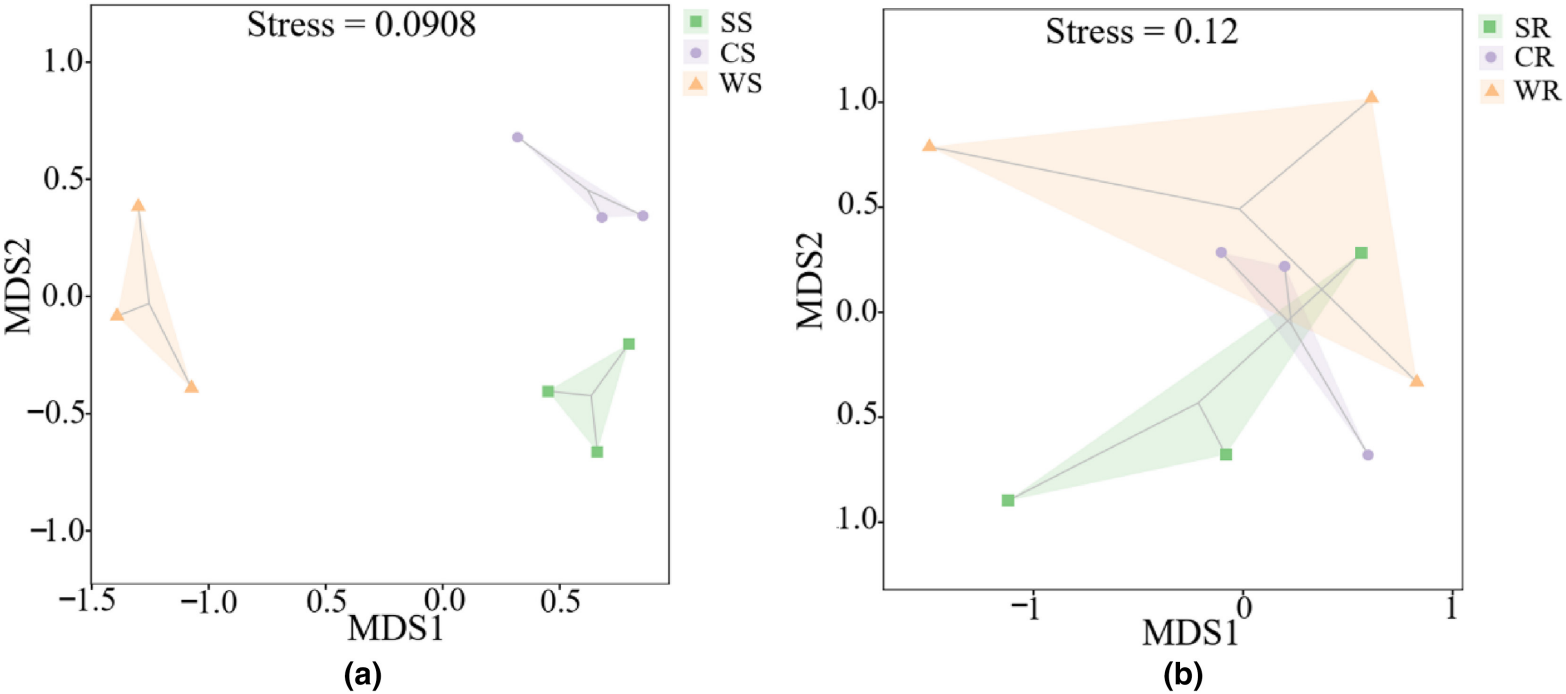
NMDS analysis of AM fungal community in rhizosphere soils and roots of *P. australis* under different water conditions. (a) Rhizosphere soils; (b) roots.

The differences of AM fungal community structure between different water conditions were further analyzed and compared using NMDS and Permanova analysis. NMDS analysis showed a clear clustering of AM fungal communities in rhizosphere soils of *P. australis* under different water conditions (Figure 7a). But there was no clear separation of the AM fungal communities in roots among different water conditions (Figure 7b). According to the results of Permanova analysis, the composition of AM fungal community in *P. australis* roots was significantly different from that in rhizosphere soil (*Pseudo‐F* = 4.59, *p* = .001). In rhizosphere soils, the composition of AM fungal community varied under different water levels (*Pseudo‐F* = 2.44, *p* = .004), whereas in roots, AM fungal community composition did not vary significantly under different water conditions (*Pseudo‐F* = 1.35, *p* = .158).

### 3.6 Relationships between AM fungal community composition and rhizosphere soil properties

**FIGURE 8:**
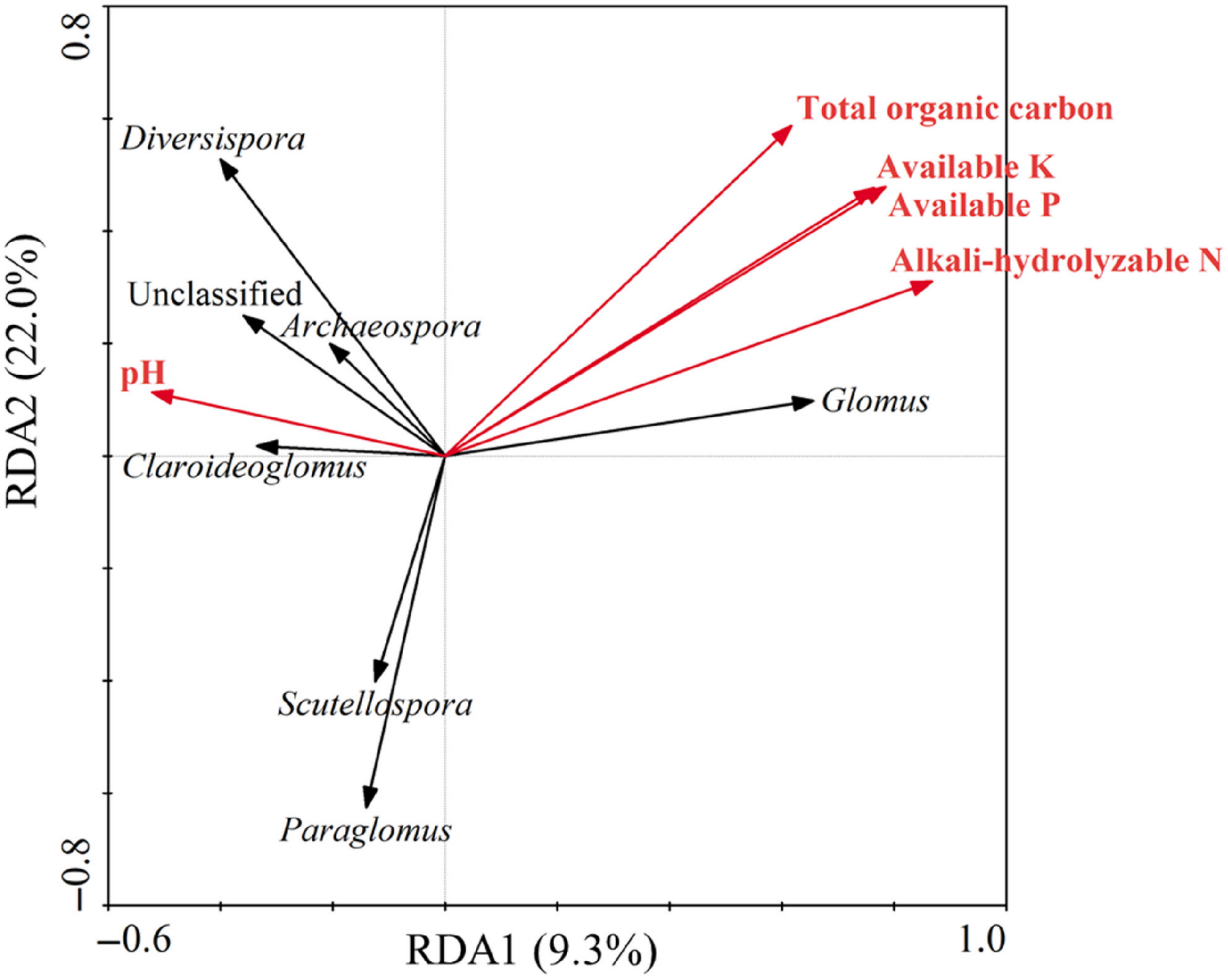
Redundancy analysis (RDA) biplot depicting the relationships between AM fungal community composition and soil properties.

Based on the above results, the AM fungal genera with obvious changes in rhizosphere soils were selected, and their relationships with soil environmental factors were analyzed by RDA. The canonical axes of first and second showed 9.3% and 22% of data variance, respectively. Results showed that the abundance of *Glomus* was positively correlated with soil alkali‐hydrolyzable N, available K, available P, and total organic carbon, but negatively correlated with soil pH. The abundances of *Paraglomus* and *Scutellospora* were negatively correlated with soil alkali‐hydrolyzable N, available P, available K, and total organic carbon, but had no obvious correlation with soil pH. The abundance of *Claroidoglomus* was positively related to soil pH, and negatively correlated with soil available K, available P, alkali‐hydrolyzable N, and total organic carbon. The abundances of *Diverspora* and *Archaeospora* were positively related to soil pH, but did not have an obvious correlation with soil alkali‐hydrolyzable N, available K, available P, and total organic carbon (Figure 8).

## 4 DISCUSSION

This study found that the content of alkali‐hydrolyzable N in flooded area was higher than that in dry area and alternating wet and dry area. This may be attributed to the timing of sampling (November). The higher biomass and litter contents of *P. australis* in autumn may result in a higher nutrient content in sediment in flooded area.

In many reported studies, the dominant genus detected in plant roots was mainly *Glomus* (Appoloni et al., 2008; Song et al., 2019; Varela‐Cervero et al., 2015). Inconsistently, *Paraglomus* was detected as the dominant genus in the roots of *P. australis* in our study. Moreover, the number of identified genera and unique OTU were higher in rhizosphere soils than those in roots. These results confirmed the partner selectivity between plants and AM fungi. AM fungal community in soils represents a species pool, and plants can freely recruit certain species (Bainard et al., 2011; Torrecillas et al., 2012). Besides, we found that the diversity of AM fungi in rhizosphere soils was higher in flooded area, but lower in alternating wet and dry area. While the diversity of AM fungi in roots was higher in dry area, but lower in flooded area, and similar with the colonization rate of AM fungi. This indicated that the water regime was the driving force for the AM fungal colonization. AM fungi exhibited strong resistance to water floods in rhizosphere soils, presumably because the influence of water floods on AM fungal propagules (resting spores) in soils was not significant due to their resistance to an adverse environment (Xu et al., 2016). Furthermore, AM fungi might resist flooding by concentrating oxygen in rhizosphere (Cooke & Lefor, 1998). However, unlike soil condition, waterflooding could directly affect roots morphology and physiology, leading to a progressive decrease in tissues available for AM fungal colonization (Vallino et al., 2014; Xu et al., 2016). In addition, considering a degree of partner selectivity and compatibility of plant‐AM fungi, the *P. australis* might select for a subset of the available pool of AM fungal taxa that could tolerate high water or low oxygen. So the species richness of AM fungi in roots was lower than that in rhizosphere soils in flooded area.

A water regime‐dependent shift in the AM fungal community in rhizosphere soils was observed in this study. In rhizosphere soils, *Diversispora*, *Scutellospora*, and *Ambispora* were more abundant in dry area, while *Glomus* preferred flooded area, and *Paraglomus*, *Claroideoglomus*, and *Archaeospora* showed preferences for alternate dry–wet conditions. This indicated that different AM fungal species were not physiologically equivalent in their tolerance to water level, and they reacted differently upon the changes of water regime in wetlands. The changes in the water regime had a stronger impact on AM fungal community in rhizosphere soils, but less on the roots. The reason may be that AM fungal community in roots was mainly affected by the physiological activities of individual plants, but less affected by the external environment of soil (Ji et al., 2020). Soil is the main carrier of AM fungi, and its physical and chemical properties have a direct impact on the survival status of AM fungi (Han et al., 2023). Based on RDA analysis, the shifts in AM fungal community structure also due to soil properties. Specifically, pH was negatively correlated with the dominant genus *Glomus*. It has been confirmed that AM fungi species required different pH ranges for development, and pH was selective for AM fungi species, and the alkaline soil was not conducive to the formation and development of the spores of *Glomus* (Costa et al., 2013; Postma et al., 2007). In addition, alkali‐hydrolyzable N was mostly positively correlated with *Glomus*. This may be because a large amount of inorganic N must be involved when AM fungi produced spores. Most studies have revealed the importance of environmental factors in shaping AM fungal communities in other ecosystems (He et al., 2016; Landis et al., 2004; Mohammad et al., 2003; Yang et al., 2021). Our study demonstrated that the water level and soil nutrients combined to drive the structure of AM fungal community in rhizosphere soils of *Phragmites australis* in wetlands.

## 5 CONCLUSION

This study identified the responses of AM fungal community to hydrological gradients in riparian *P. australis* wetlands. Soil properties and AM fungal community in rhizosphere soils exhibited variation under different hydrological gradients, but AM fungal community in roots was not significantly affected. The flooded condition accumulated more soil nutrients and stimulated the increase of AM fungal Chao1 richness in rhizosphere soils. Hydrological conditions and available nutrient levels jointly led to the changes of AM fungal composition in soils. These results promote our understanding on the effects of hydrological gradients on shaping AM fungal communities in wetlands. However, due to the limited sampling range in this study, future studies should continue to explore the relationships between AM fungal colonization, function, and environmental factors across at a regional scale in wetland ecosystems.

## AUTHOR CONTRIBUTIONS

**Xue‐dong Chen:** Conceptualization (lead); data curation (equal); formal analysis (equal); writing – original draft (lead); writing – review and editing (equal). **Ying Zhu:** Methodology (equal); visualization (equal); writing – review and editing (equal). **Mei‐na Feng:** Methodology (equal); visualization (equal); writing – original draft (equal). **Ji‐hang Li:** Methodology (equal); visualization (equal); writing – original draft (equal). **Ming‐yan Shi:** Conceptualization (equal); supervision (equal).

## CONFLICT OF INTEREST STATEMENT

The authors have no conflict of interest to declare.

## Supporting information


Figures S1–S3.

## Data Availability

All raw sequences were deposited in the NCBI Sequence Read Archive under accession number SRP440790.
