# Supplementary material for: Community responses of arbuscular mycorrhiza fungi to hydrological gradients in a riparian Phragmites australis wetland
Source: Ecol Evol. 2024 Apr 11;14(4):e11271. doi: 10.1002/ece3.11271 (PMC11009486; doi:10.1002/ece3.11271)
Supplement: Supplementary file 1 — Figures S1–S3. [file ECE3-14-e11271-s001.docx]

**Supplementary Materials**

Community responses of arbuscular mycorrhiza fungi to hydrological gradients in a riparian *Phragmites australis* wetland

Xue-dong Chen^1,2*^, Ying Zhu^1^, Mei-na Feng^1^, Ji-hang Li^1^, Ming-yan Shi^1,2^

^1^ College of Life Science, Luoyang Normal University, Luoyang 471934, Henan, China

^2^ West Henan Yellow River Wetland Ecosystem Observation and Research Station, Luoyang Normal University, Luoyang 471934, Henan, China

^*^**Correspondence**: Xue-dong Chen, College of Life Science, Luoyang Normal University, Luoyang 471934, Henan, China.

Email: chenxuedong1224@163.com


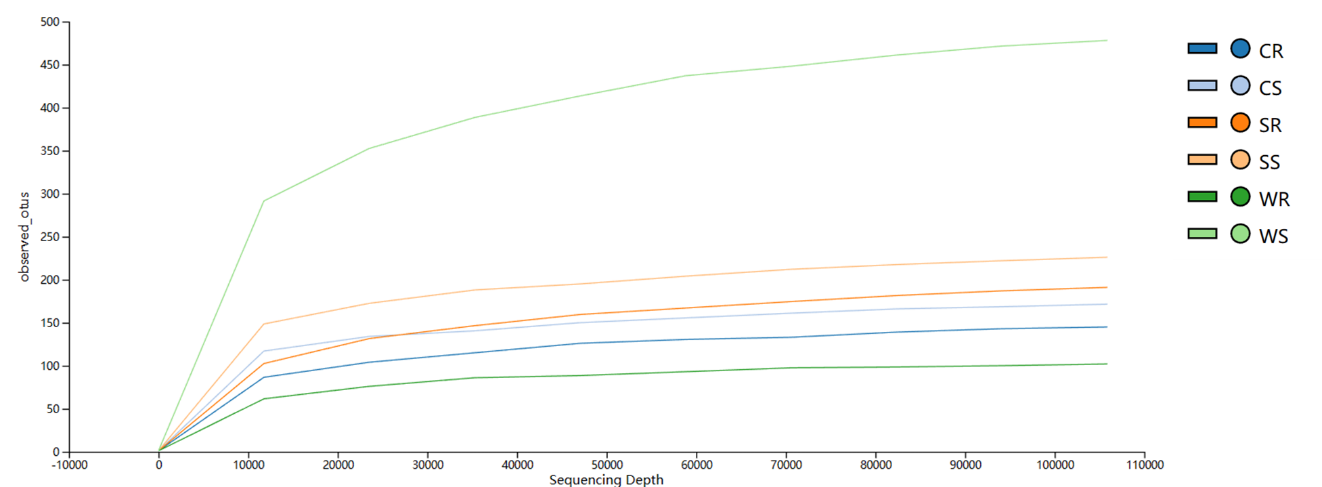


FIGURE S1 Rarefaction curves.

Note: SS: rhizosphere soils of *P. australis* in dry area; CS: rhizosphere soils of *P. australis* in alternating wet and dry area; WS: rhizosphere soils of *P. australis* in flooded area; SR: roots of *P. australis* in dry area; CR: roots of *P. australis* in alternating wet and dry area; WR: roots of *P. australis* in flooded area.


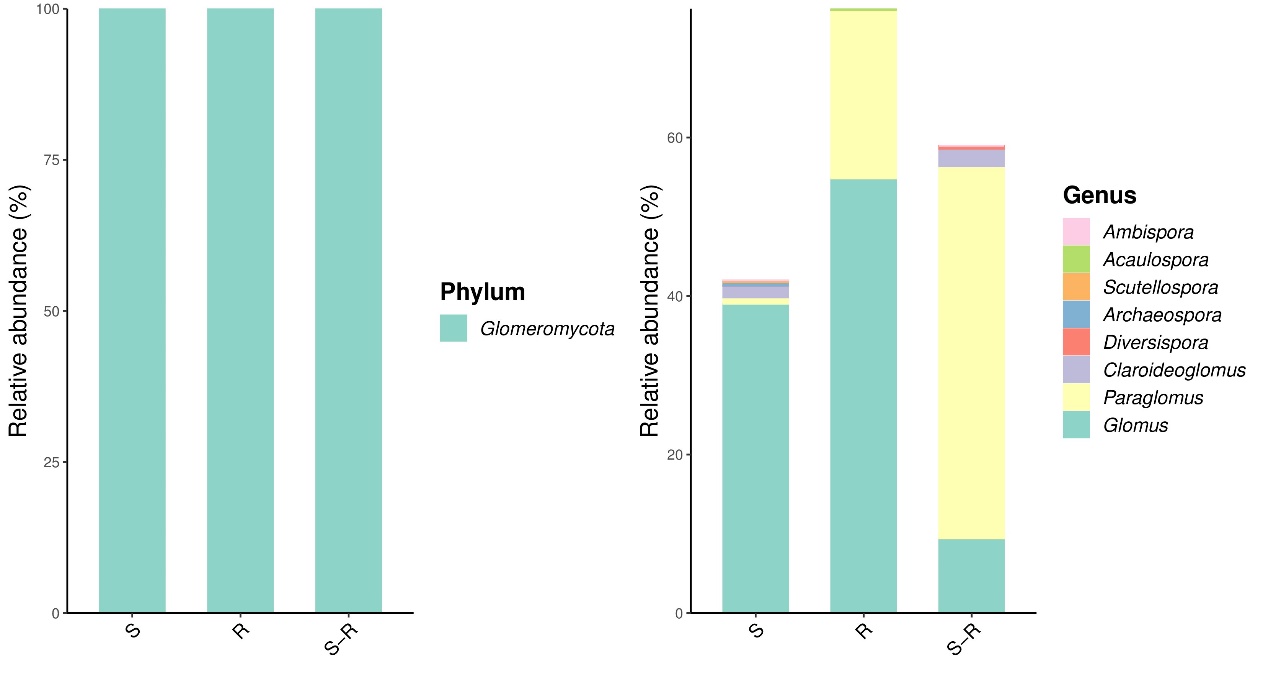


FIGURE S2 The species abundance composition of each region in the venn diagram. The horizontal axis represents the OTU sets corresponding to different regions of the venn diagram, and the vertical axis represents the relative abundance of different phyla (left) and genera (right).

Note: S: the region with unique OTUs in rhizosphere soils; R: the region with unique OTUs in roots; S-R: the region with shared OTUs between rhizosphere soils and roots.





FIGURE S3 The relative abundance of *Glomus* and *Paraglomus* in roots and rhizosphere soils of *P. australis* under different water conditions. Bars are means ± SE.
